# 

**Published:** 1884-05-20

**Authors:** 


Entered at the Post-Office at Atlanta, as second-class matter.

VOL. XIV. MAY 20, 1884. NO. 5.

### THE SOUTHERN MEDICAL RECORD:

### A MONTHLY JOURNAL OF PRACTICAL MEDICINE.

Terms: $2 00 per Annum, in Advance; 4 Copies $6.00; Single Copies 20 Cents.

*"QUICQUID PRÆCIPIES ESTO BREVIS."*

### WHERE TO BUY.

Fellows' Hypo-Phos-Phites........ 1
Natural Uterine Supporter........ 2
Galvanic & Faradic Battery,........ 3
Colden's Liquid Beef Tonic........ 4
Woolrich & Co........ 5
Moore's Business University........ 5
Horlick's Food for Infants and Invalids, 5
T. B. Wheeler, M. D........ 6
Syrup Hypophos. Comp. C. P........ 6
Warren's Food Flour........ 7
Geo. P. Rowell & Co........ 7
Imperial Granum........ 8
French Wine Coca........ 9
Cincinnati Sanitarium........ 9
Battle & Co........ 10
University of Pennsylvania........ 11
College for Med. Practitioners........ 11
Reed & Carnrick........ 12
Harter's Iron Tonic........ 13
Richardson & Co........ 14
Lactopeptine........ 15
Academic Pharmaceutic Company........ 16
Lambert & Co........(colored leaves)
H. C. Hall........(colored leaves.)
Hall & Ruckel........(colored leaves.)
Landrum & Litchfield......(colored leaves.)
A. A. Mellier........(colored leaves)
Sharp & Dohme........(Inside front cover)
Reed & Carnrick,........(inside back cover)
Parke, Davis & Co.....(Outside back cover)

Address R. C. WORD, M.D., Managing Editor, Atlanta, Ga.

